# Robustness of Empirical Vibration Correlation Techniques for Predicting the Instability of Unstiffened Cylindrical Composite Shells in Axial Compression

**DOI:** 10.3390/polym12123069

**Published:** 2020-12-21

**Authors:** Eduards Skukis, Gints Jekabsons, Jānis Andersons, Olgerts Ozolins, Edgars Labans, Kaspars Kalnins

**Affiliations:** 1 Institute of Materials and Structures, Riga Technical University, 6A Kipsalas str., LV-1048 Riga, Latvia; edskukis@gmail.com (E.S.); gints.jekabsons@rtu.lv (G.J.); olgerts.ozolins@rtu.lv (O.O.); 2 Institute for Mechanics of Materials, University of Latvia, 3 Jelgavas str., LV-1004 Riga, Latvia; 3 Faculty of Aerospace Engineering, Delft University of Technology, 2629HS Delft, The Netherlands; edgars.labans@gmail.com; 4 Ikskile Centre of Composite Competence Ltd., LV-5015 Ikskile, Latvia

**Keywords:** polymer composite, buckling, imperfection, vibration correlation technique, natural frequencies

## Abstract

Thin-walled carbon fiber reinforced plastic (CFRP) shells are increasingly used in aerospace industry. Such shells are prone to the loss of stability under compressive loads. Furthermore, the instability onset of monocoque shells exhibits a pronounced imperfection sensitivity. The vibration correlation technique (VCT) is being developed as a nondestructive test method for evaluation of the buckling load of the shells. In this study, accuracy and robustness of an existing and a modified VCT method are evaluated. With this aim, more than 20 thin-walled unstiffened CFRP shells have been produced and tested. The results obtained suggest that the vibration response under loads exceeding 0.25 of the linear buckling load needs to be characterized for a successful application of the VCT. Then the largest unconservative discrepancy of prediction by the modified VCT method amounted to ca. 22% of the critical load. Applying loads exceeding 0.9 of the buckling load reduced the average relative discrepancy to 6.4%.

## 1. Introduction

Carbon fiber-reinforced polymer (CFRP) composites possess highly competitive specific strength and stiffness properties. Moreover, the properties of CFRP laminates can be tailored to the expected loading conditions by optimizing their lay-up. The excellent strength-to-weight and stiffness-to-weight ratios, combined with controlled anisotropy of mechanical properties, has ensured a widening application of CFRPs. These characteristics of CFRPs are particularly beneficial in weight-conscious sectors of industry, such as aerospace (see, e.g., [1,2]).

For thin-walled CFRP structures subjected to compressive service loads, the failure mode limiting their load-bearing capacity is, typically, the loss of stability. Estimates of the buckling load based on an idealized shell geometry and loading are known to be unconservative due to imperfection sensitivity of the onset of instability, especially pronounced for monocoque (unstiffened) shells. In the design phase, the effect of imperfections on the buckling load can be allowed for via a knock-down factor (KDF), i.e., the ratio of the actual critical load of imperfect shell, *P*_b_, and the predicted buckling load of a perfect shell, *P*_cr_, estimated according to the linear bifurcation theory. Such empirical lower-bound KDFs for cylindrical shells have been established in 1960s based on accumulated experimental data [3], and later specified for anisotropic, laminated composite cylinders in axial compression [4]. An alternative approach of determining KDF for conservative design is based on the nonlinear numerical modelling of buckling in the presence of an assumed worst-case imperfection in the composite cylinder. Such a worst-case situation can be presented by, e.g., an appropriately scaled linear buckling mode-shaped imperfection, a geometric dimple-shaped imperfection, a single boundary perturbation, a single perturbation load, axisymmetric imperfections, scaled mid-surface imperfections, or the membrane stiffness reduction, as described in [5,6,7,8,9]. Since the characteristics of imperfections can be considered as random variables [10], probabilistic methods are also applied in the shell buckling analysis, leading to probabilistic design approaches [8,9,10,11]. Deterministic and probabilistic methods providing lower-bound estimate of KDF ensure safe, but not necessarily efficient, design of shells.

The accuracy of buckling load prediction for an already produced shell can be further increased using additional information on its specific individual characteristics obtainable by nondestructive means of inspection. Imperfections in composite shells can be of geometrical, structural, material nature, or related to loading of the shell. When an imperfection signature of a shell can be determined in sufficient detail, a nonlinear numerical buckling analysis allowing for imperfections provides a close estimate of the buckling load [12,13,14,15]. Notably, shells sensitive to geometrical imperfections also exhibit sensitivity to load imperfections [16]. It precludes neglecting either group of imperfections, although the relative contribution of different types of imperfections to facilitating the onset of instability may vary [12,15,17]. The incorporation of measured initial geometric and thickness imperfections, thickness-adjusted material property variations, measured loading imperfections, elastic radial support conditions, and allowing for selected specimen parameter uncertainties (e.g., uncertainty in the imperfection measurement accuracy, fiber and matrix properties, fiber volume fraction, etc.) in shell models enabled an accurate prediction of the compression response of unstiffened thin-walled graphite-epoxy cylindrical shells [13]. However, such a detailed characterization of a shell may appear impractical in most industrial applications.

A viable alternative to measuring imperfections, with a subsequent numerical analysis of their effect on stability, is the nondestructive testing of a shell. In this way, the integral effect of imperfections on its eigenfrequencies is established and the buckling load is evaluated by the vibration correlation technique (VCT). The VCT approach implies experimentally determining the reduction in the natural frequency of a structural element with increasing compressive applied load and estimating the buckling load based on such data. Simply-supported imperfection-insensitive structural elements, for which the vibration and buckling modes coincide, exhibit a linear relation between the applied load *P* and the first natural frequency *f*_m_ squared (see., e.g., [18,19])

(1) $$\frac{P}{P_{b}}+{\left(\frac{f_{m}}{f_{0}}\right)}^{2}=1$$

where *P*_b_ is the buckling load and *f*_0_ denotes the respective natural frequency at *P* = 0. Having measured the variation of the natural frequency with load in the range of relatively low loads, Equation (1) can be applied to estimating the buckling load as *P*_b_ = *P* at *f*_m_ = 0. However, imperfection-sensitive plates and shells exhibit a much steeper reduction in the natural frequency when the applied load approaches the actual *P*_b_ than that implied by Equation (1), which leads to an unconservative prediction for the buckling load.

A number of empirical and semi-empirical modified VCTs have been proposed to reflect the effect of imperfections on the load–natural frequency relation of shells, as recently reviewed in, e.g., [20,21,22]. The most accurate and extensively studied VCT method for unstiffened cylindrical shells, proposed in [23], departs from the common approach of relating the onset of buckling to fading of the natural frequency of the shell. Instead, it is suggested that buckling takes place when (1 − *P*/*P*_cr_)^2^, assumed to be a second-order polynomial function of 1 − (*f*_m_/*f*_0_)^2^, reaches its minimum. A graphical interpretation of the procedure is as follows [23]: the experimental load–natural frequency data are presented as a plot of (1 − *P*/*P*_cr_)^2^ versus 1 − (*f*_m_/*f*_0_)^2^, the data points are approximated by fitting a second-order polynomial, the minimum value of this polynomial, *ξ*^2^, is found, and the VCT estimate of the buckling load *P*_VCT_ is obtained from the equality 1 − *P*_VCT_/*P*_cr_ = *ξ*. The described approach has also been validated numerically [24], although only considering one specific imperfection group, namely, geometrical imperfections.

This VCT method has been applied for estimation of the buckling load in axial compression of CFRP shells of various lay-ups [21,23,25,26,27,28], including a variable-angle tow CFRP shell [28], as well as grid-stiffened glass/epoxy [29], composite lattice sandwich [22], stainless steel [26] and aluminum alloy [30,31] shells. Apart from a pure axial loading, the critical load for pressurized aluminum alloy shells [31] has also been studied. Notably, a numerical simulation of the experimental VCT procedure of shells, implementing either real measured imperfections or appropriate assumed imperfections in the shell model, has also produced encouraging results [32].

Each of the methods for estimating the critical load discussed above has its specific advantages and conditions of application. In the design stage, the buckling load of a shell can be evaluated only by the KDF approach, either based on accumulated data [3,4] or a worst-case imperfection analysis [5,6,7,8,9,10,11]. Since a lower-bound estimate of KDF is determined in this way, the critical load is underestimated, leading, possibly, to overdesigning of the shell. When an actual shell is produced, its imperfection signature can be determined, or vibration tests conducted, in order to characterize the specific shell. The advantage of imperfection characterization with a subsequent numerical modelling of instability onset [12,13,14,15] is that no mechanical tests of the shell are needed. However, a sufficiently detailed characterization of shell imperfections may be overly complicated in industrial applications. The advantage of VCT is that only a relatively uncomplicated nondestructive mechanical test of a shell is required [20,21,22,23,24,25,26,27,28,29,30,31] instead of a detailed inspection of different imperfections of the shell. However, the boundary conditions of the structural element have to be accurately recreated in the shell test. Another limiting factor of applying the VCT is disappearing of the monitored mode shapes, sometimes observed at increasing load levels [28].

Evaluation of the applicability of VCT [23] has not been conclusive yet, partially due to rather limited testing programs—only a few monocoque CFRP shells were tested in each of the studies [21,23,25,26,27,28]. Although most works report close conservative estimates of the buckling load by the VCT, overestimations are also revealed in some studies, e.g., in [27], where buckling loads nonconservative by about 5 and 8% were found by the VCT for the two cylinders tested. Therefore, a more extensive testing appears necessary to elucidate the accuracy and robustness of the VCT.

With such an aim, a wider test program has been initiated; in the current study, 21 cylindrical CFRP shells of the same lay-up, but four different diameter and height combinations, have been produced and tested. Their response has been analyzed by means of the VCT technique [23] and by an empirical modification of the latter. The contribution of the present study to the experimental and analytical development of VCT comprises:

- presenting results of one of the largest test programs to-date concerning the application of VCT for evaluating the axial buckling load of unstiffened cylindrical CFRP shells;
- proposing an empirical modification of the existing VCT analysis that increases the accuracy of estimates of the buckling load;
- evaluating the robustness of VCT with respect to variations in shell geometry, mounting and loading methods, and the preload.

The results obtained suggest that the vibration response from the unloaded state of the shell up to loads exceeding 0.25 of the linear buckling load needs to be characterized to enable a reliable VCT prediction. The largest discrepancy in overestimation of the critical load by VCT was limited to about 22% when the modified VCT method was applied using the load range mentioned. The accuracy of the VCT was found to be virtually insensitive to shell geometry, the method of load introduction, and the absence of natural frequency data for the unloaded shell.

## 2. Empirical VCT Techniques

The VCT method for unstiffened cylindrical shells proposed in [23] assumes the relation between the natural frequency *f*_m_ of the shell and the applied compressive load *P* in the form (see also [21,28])

(2) $${\left(1-p\right)}^{2}=C_{2}\;{\left(1-f^{2}\right)}^{2}+C_{1}\left(1-f^{2}\right)+C_{0}$$

where *p* denotes the applied load normalized by the linear buckling load, *p* = *P*/*P*_cr_, and *f* = *f*_m_/*f*_0_ is the ratio of natural frequencies of the shell under an axial load and in the load-free condition. The onset of instability is related to (1 − *p*)^2^ reaching its minimum, i.e., the first derivative of the function Equation (2) with respect to its argument 1 − *f*^2^ being zero,

(3) $$\frac{d{\left(1-p\right)}^{2}}{d\left(1-f^{2}\right)}=0$$

and the second derivative being positive. It follows from Equations (2) and (3) that

(4) $$Min\left[{\left(1-p\right)}^{2}\right]=\xi^{2}=C_{0}-\frac{C_{1}^{2}}{4C_{2}}$$

and *C*_2_ > 0. In Equation (4), *ξ* is interpreted as the reduction in the load-carrying capacity of a shell due to the presence of imperfections. Finally, the VCT-predicted critical load is

(5) $$P_{\mathrm{VCT}}=P_{\mathrm{cr}}\;\left(1-\xi \right)$$

The functional form of Equation (2), with *C*_2_ = *C*_0_ =1 and *C*_1_ = −2, has been noted to comply with that of a perfect simply supported shell given by Equation (1), and Equation (3) shown to provide a correct buckling condition of *f*_m_ = 0 in such a case [24]. A similar derivation for a clamped shell has been performed in [32].

**Figure 1:**
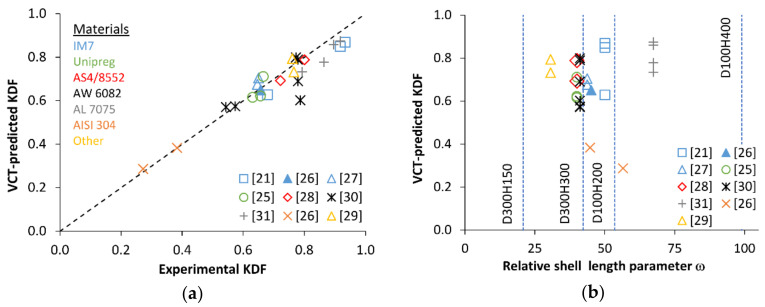
Application of vibration correlation technique (VCT) [23] to monocoque shells: (**a**) Parity plot of VCT-predicted vs. experimental knock-down factors (KDFs); (**b**) VCT-predicted KDF as a function of the relative length parameter of shell (the dashed lines indicate the composite shell geometries considered in the present study).

A comparison of applications of this VCT based on monitoring the reduction of each of the first four natural frequencies revealed that using the lowest natural frequency in Equation (2) produced the most accurate prediction for the buckling load [28]. The prediction accuracy was also found to generally improve when experimental data of *f*_m_ vs. *P* for greater *P*/*P*_b_ ratios were used, as expected. The VCT-predicted and experimental KDFs for the highest load levels used in each of the studies [21,25,26,27,28,29,30,31] are presented in a parity plot in Figure 1a. Specifically, for CFRP shells, the maximum loads in these VCT studies amounted to *P*/*P*_b_ = 0.81…0.98 [21,25,26,27,28], whereas for metal shells, they were *P*/*P*_b_ = 0.54…0.99 [26,30,31].

Further, a wide range of cylinder geometries, as characterized by the relative shell length parameter $\omega =H/\sqrt{R\cdot t}$ [33], expressed via cylinder height H, radius R, and wall thickness *t*, has been covered in the tests, Figure 1b. A very close correlation between the KDFs determined by buckling tests and by the VCT is seen in Figure 1a, with the most conservative VCT-predicted buckling load *P*_VCT_ amounting to about 76% of the experimental critical load and the most unconservative *P*_VCT_ exceeding *P*_b_ by ca. 8%.

Such a good predictive capacity is likely to stem from correctly capturing the phenomenology of the underlying physical mechanism of instability by this VCT method. Experimental studies (see, e.g., [20,23]) have indicated that shell buckling is preceded by a swift transition from the gradual reduction in the natural frequency with increasing load to a rapid drop of frequency. This has also been demonstrated by a theoretical modelling of vibrations of imperfect shells, which allowed constructing load–eigenfrequency diagrams up to the critical load [34]. Notably, instability criterion Equation (3) can also be interpreted as marking the onset of a rapid change in the natural frequency, df/dp→∞  or, equivalently, dp/df→0. Indeed, elementary transformations of the left-hand side of Equation (3) yield

(6) $$\frac{d{\left(1-p\right)}^{2}}{d\left(1-f^{2}\right)}=\frac{\frac{d{\left(1-p\right)}^{2}}{dp}dp}{\frac{d\left(1-f^{2}\right)}{df}df}=\frac{1-p}{f}\frac{dp}{df},$$

demonstrating that meeting the instability condition Equation (3) also implies that dp/df=0.

Since the form of the right-hand side of Equation (2) has been arrived at empirically [23], it appears plausible that similar functions with this property can be used to locate the instability onset. We consider an empirical modification of Equation (2) employing a second-order polynomial of 1 − *f*:

(7) $${\left(1-p\right)}^{2}=c_{2}\;{\left(1-f\right)}^{2}+c_{1}\left(1-f\right)+c_{0}$$

Then, the minimum value of (1 − *p*)^2^ as a function of 1 − *f* is given by

(8) $$\xi^{2}=c_{0}-\frac{c_{1}^{2}}{4c_{2}}$$

at *c*_2_ > 0, and the predicted buckling load is determined by Equation (5) using the value of *ξ* from Equation (8). It can be easily checked that

(9) $$\frac{d{\left(1-p\right)}^{2}}{d\left(1-f\right)}=2\left(1-p\right)\frac{dp}{df}$$

i.e., the condition dp/df=0 holds at the critical load.

## 3. Materials and Methods

In the following subsections, the material and the manufacturing method of shells are described, the methods for mounting of shells in the test rig are defined, and procedures for determining the shell thickness, buckling load, and vibration response are presented.

### 3.1. Material

A unidirectional carbon fiber/epoxy prepreg Unipreg^®^ (UNICARBON^®^, produced in Kaunas, Lithuania), with a 100 g/m^2^ nominal areal density, was used for manufacturing of cylindrical composite shells. Two batches of nominally identical prepregs were obtained from the producer in consecutive separate shipments. For clarity and traceability, the letter N was included in identifiers of the shells made using prepreg of the latest batch.

The elastic properties of a unidirectionally reinforced (UD) composite produced from the prepreg are presented in Table 1.

### 3.2. Manufacture of Shells

Three-ply cylindrical shells of lay-up [0/45/−45] (angles were measured with respect to the cylinder axis, starting from the innermost ply) were produced by hand lay-up using steel mandrels. The shells were consolidated by means of vacuum bagging and cured at elevated temperatures. The cure cycle consisted of subjecting the shells to an 80 °C temperature for 1 h followed by 3 h at 130 °C. In this way, shells of nominal diameter D = 100 mm and two different heights, H = 200 and 400 mm, as well as shells with D = 300 mm and H = 150 and 300 mm, were manufactured.

### 3.3. Tests

#### 3.3.1. Characterization of Shell Thickness

The variation of thickness for each shell was determined by an ultrasonic technique. To enable an efficient scanning of cylinders, a Hilgus USPC 3010 HF equipment (Hillger NDT GMBH, Braunschweig, Germany) was modified to replace the *y*-coordinate drive by a rotational drive. A support frame for cylinders was made of an aluminum profile system with front and rear axles, a lathe chuck on the drive end, and a PLA6 cone passive end. During ultrasonic scanning, the cylindrical shells were supported on machined foam end plates.

#### 3.3.2. Shell Buckling Tests

A universal Zwick 100 quasi-static testing machine (Zwick GMBH, Ulm, Germany) was used for axial compression tests of the composite cylinders.

**Figure 2:**
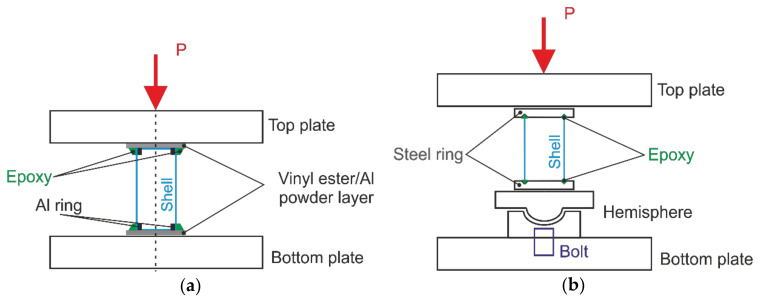
Schematic of the setup for compression tests of composite cylinders: (**a**) Testing by means parallel loading plates; (**b**) Testing using a hemispherical joint.

Two methods of load introduction, shown schematically in Figure 2, were applied. The majority of the cylinders were tested employing horizontal parallel steel plates, with a compression plate rigidly bolted to the machine crosshead, Figure 2a. The loading was displacement-controlled, at a rate of 1 mm/min. The load was measured by a single load cell located between the loading plate and the respective crosshead of the loading frame, while the shortening was determined as the average of readings of three LVDTs placed around the shell at 120° angular intervals.

The rest of specimens were tested as shown schematically in Figure 2b. Specifically, a hemispherical joint was installed on the bottom plate, enabling rotation of the supporting plate, thus eliminating bending moments and, hence, promoting self-alignment of the cylinder axis with the loading direction. For this loading set-up, the shortening of specimens was measured via the crosshead displacement.

**Figure 3:**
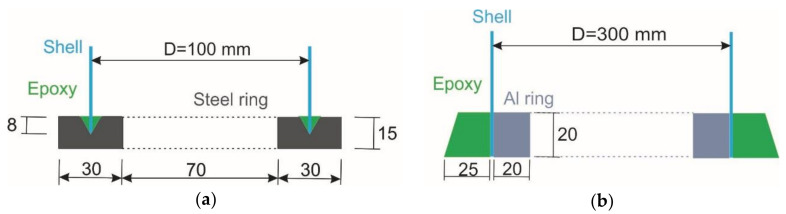
Schematic of the mounting of composite cylinders: (**a**) In the groove of a steel ring; (**b**) Potting onto plates of the test machine. Dimensions in the figure are in mm.

Edges of the 100-mm-diameter shells were mounted between parallel steel rings, in 8 mm deep circular grooves with a V-shaped cross section, which were filled with a mixture of epoxy resin and fine sand, as schematically presented in Figure 3a. Specimens with such a mounting were tested either employing a hemispherical joint, Figure 2b, or placing them between the parallel steel plates of the test machine. 

The top and bottom edges of the 300-mm-diameter shells were both clamped by aluminum rings from the inside and potted with an epoxy mortar containing fine sand and slag, Figure 3b. The shells were mounted directly unto the loading plates, as shown in schematic Figure 2a, filling the narrow gaps between the specimen and the plates by a fast-curing alumina powder-filled vinyl ester resin in order to mitigate the effects of contact surface unevenness, which would result in loading imperfections.

The buckling mode shapes of the cylinders were captured photographically.

#### 3.3.3. Characterization of Vibration Response

**Figure 4:**
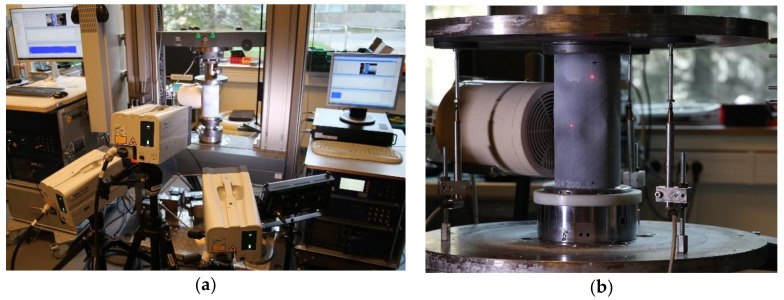
Test setup for characterization of the vibration response of a carbon fiber-reinforced polymer (CFRP) cylinder: (**a**) Overview of the experimental setup with a specimen installed between loading plates; (**b**) Placement of the loudspeaker for excitation of vibrations.

The natural frequencies and vibration modes were determined using a Polytec laser vibrometer (Polytec GMBH, Waldbronn, Germany); the test setup is shown in Figure 4.

A grid of points distributed on a small area of the cylinder was scanned. The area with 600 grid points for scanning was found to yield a sufficiently detailed modal response. For excitation of vibrations, a loudspeaker was used. It was placed perpendicularly to the measurement zone on the opposite side of the specimen, Figure 4b. The measurements were conducted within the frequency range from few hundreds of Hz, well below the lowest natural frequency, to ca. 1600 Hz to ensure that more than ten first natural vibration modes were covered. The vibration response was characterized at 9 to 18 load levels, ranging from the unloaded state to more than 90% of the buckling load.

## 4. Results and Discussion

### 4.1. Buckling Loads and Modes

**Figure 5:**
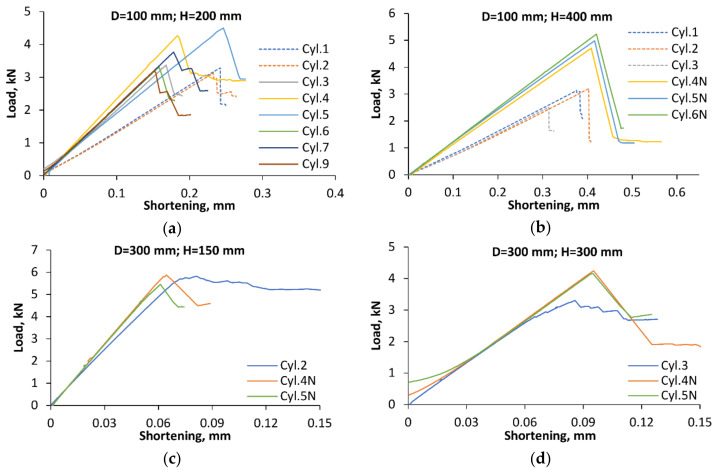
Load–shortening diagrams of cylinders with dimensions: (**a**) D = 100 mm and H = 200 mm; (**b**) D = 100 mm and H = 400 mm; (**c**) D = 300 mm and H = 150; (**d**) D = 300 mm and H = 300. Results of tests employing a hemispherical joint are plotted by dashed lines, for parallel plates—by solid lines.

Axial compression tests of CFRP cylinders were performed as described in Section 3.3.2, and the load–shortening diagrams obtained are presented in Figure 5. 

The largest number of cylinders tested had a diameter of 100 mm and height of 200 mm; they were produced from the same prepreg batch and shared the same mounting technique. The specimens were tested either employing parallel loading plates, as shown schematically in Figure 2a, or a hemispherical joint (Figure 2b). It is seen in Figure 5a that the loading method markedly affected the apparent stiffness and the instability onset of shells. Specifically, the specimens tested using a hemispherical joint (Cyl. 1 and 2) exhibited lower apparent stiffness and buckled at lower loads than the nominally identical specimens (Cyl. 3 to 9) placed between parallel loading plates. The critical load data obtained are presented in Table 2. The variability of the apparent stiffness among the specimens sharing the same load introduction method is likely to be caused by fiber alignment and potting geometry variations, as well as by the scatter in shell thickness.

For shells of the same 100-mm diameter but a larger height of 400 mm, an additional factor of variability was manufacturing the specimens from two batches of a nominally identical prepreg material. The letter N in the cylinder number is used to identify the shells made from prepreg of the latest batch. In this case, the apparent stiffness and critical load of the cylinders tested using a hemispherical bearing (Cyl. 1 to 3 in Figure 5b) also appeared to be consistently lower than that obtained via parallel-plate tests (Cyl. 4N to 6N). Since this trend held both for single-batch shells, Figure 5a, and for shells produced from different batches, Figure 5b, we tentatively conclude that variations in the prepreg properties between these batches, if any, can be neglected in the buckling analysis.

The shells of 300-mm diameter were produced from prepregs coming from either of the two batches, but mounted and tested in the same way. The load–shortening diagrams in Figure 5c,d suggest that the prepreg batch used for specimen production had no apparent systematic effect on either the buckling load or the apparent stiffness of shells.

**Figure 6:**
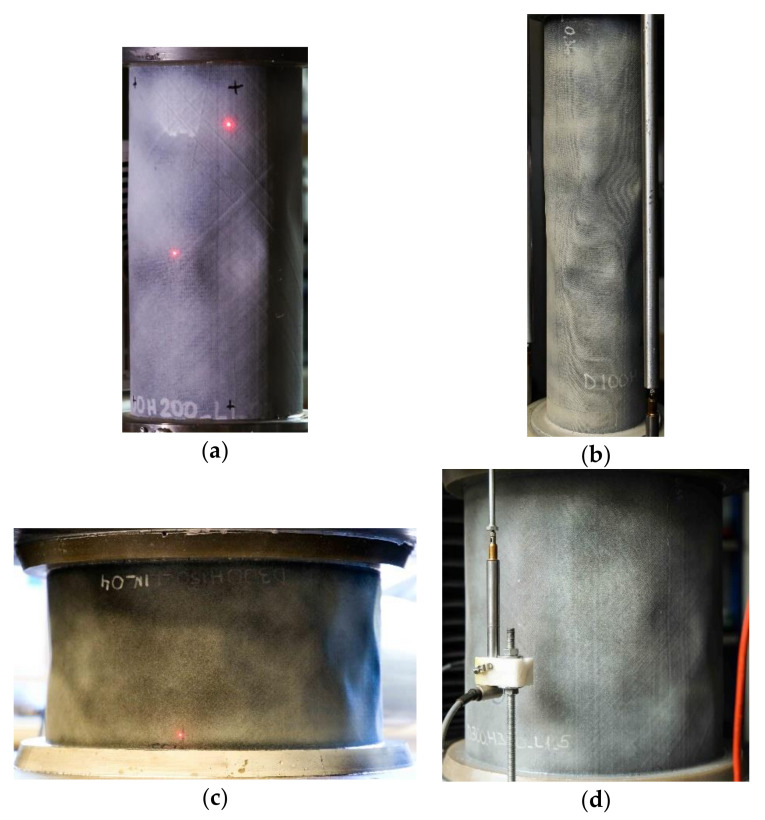
Buckling modes of cylinders with the following dimensions: (**a**) D = 100 mm and H = 200 mm; (**b**) D = 100 mm and H = 400 mm; (**c**) D = 300 mm and H = 150; (**d**) D = 300 mm and H = 300.

The buckling modes of shells can be discerned in the photographs in Figure 6.

### 4.2. Vibration Spectra and Modes

**Figure 7:**
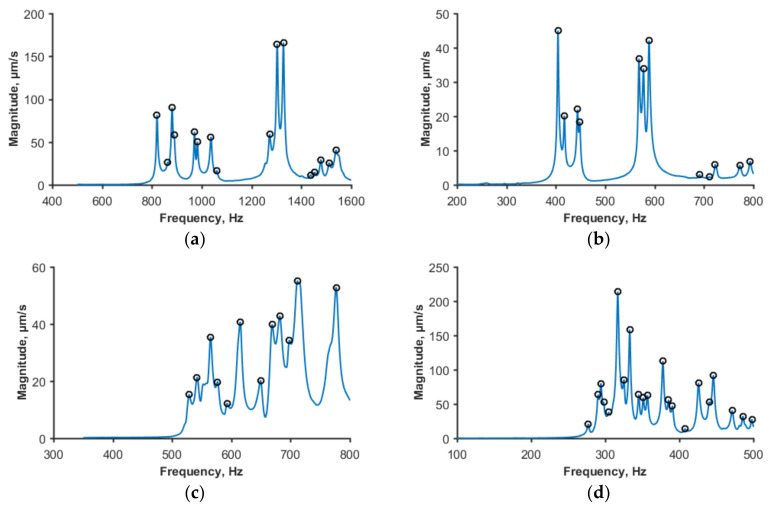
Typical natural frequency spectra of unloaded cylinders of dimensions: (**a**) D = 100 mm and H = 200 mm; (**b**) D = 100 mm and H = 400 mm; (**c**) D = 300 mm and H = 150; (**d**) D = 300 mm and H = 300.

The application of VCT implies identification of the first (or one of the lowest) natural frequency of a shell and experimentally determining the reduction in this frequency with increasing compressive load. With this aim, the natural frequency spectra of the shells were measured as described in Section 3.3.3, starting from load-free conditions. An exception was the cylinders tested using a hemispherical joint, see the schematic in Figure 2b. For these specimens, a small controlled compressive load *P*_min_ (in the range of 0.14 to 0.25 kN) was applied instead of *P* = 0 to reliably fix the specimen in position when characterizing the initial natural frequency spectrum. Typical spectra for the shells of each geometry considered are shown in Figure 7. Vibration characteristics of the shells are seen to differ, as expected, reflecting differences in their geometry. The lowest eigenfrequency of each of the cylinders was determined from the relatively densely populated low-frequency part of the spectrum.

**Figure 8:**
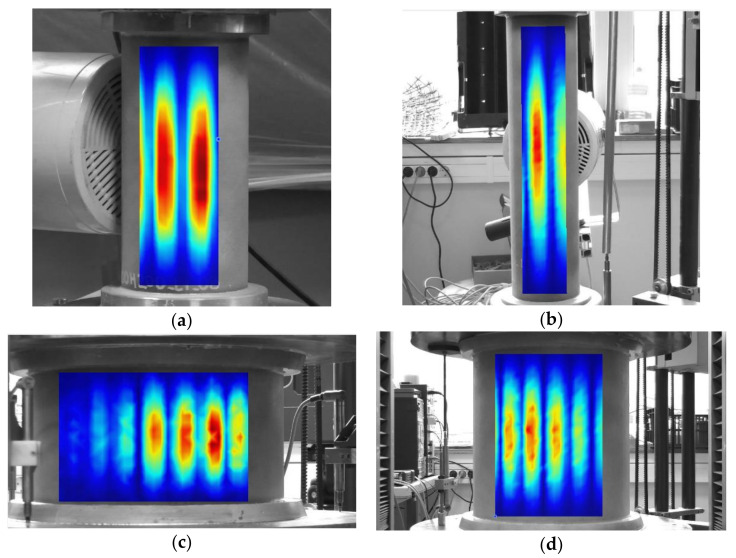
Mode shapes corresponding to the first natural frequency of unloaded cylinders of dimensions: (**a**) D = 100 mm and H = 200 mm; (**b**) D = 100 mm and H = 400 mm; (**c**) D = 300 mm and H = 150; (**d**) D = 300 mm and H = 300.

Vibration mode shapes for the first natural frequency are presented in Figure 8. The vibration modes obviously differed from the shell buckling modes seen in Figure 6, as expected for thin-walled unstiffened cylinders. For all the shell geometries considered, the first vibration mode exhibited one longitudinal half-wave, *n* = 1, while the number of circumferential half-waves, *m*, depended on shell geometry. The circumferential half-wave number was estimated as in [28], by measuring the angular size *φ* of a single half-wave and evaluating the integer part of the ratio 360°/*φ*. The characteristics (*m*, *n*) obtained from mode shapes captured by vibrometer, Figure 8, where as follows: (12, 1) for 200-mm and (6,1) for 400-mm height cylinders with a 100-mm diameter; (32, 1) for 150-mm; and (22, 1) for 300-mm height cylinders with 300-mm diameter. No change in the half-wave numbers (*m*, *n*) was observed with increasing load.

**Figure 9:**
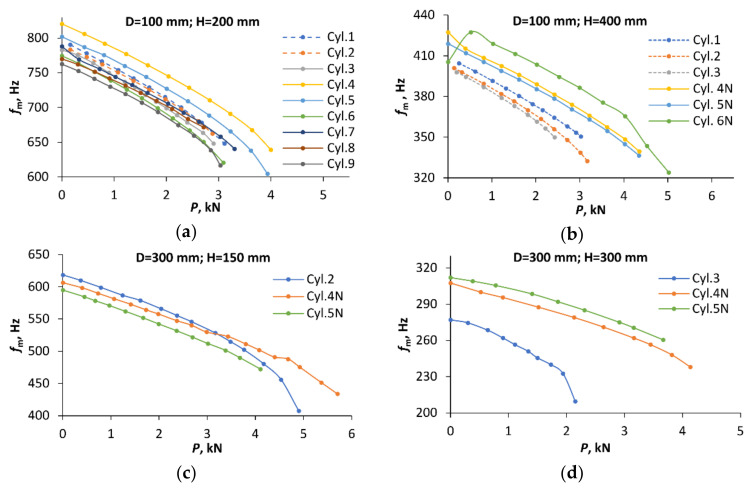
Reduction of the natural frequency due to the axial compression for cylinders of dimensions: (**a**) D = 100 mm and H = 200 mm; (**b**) D = 100 mm and H = 400 mm; (**c**) D = 300 mm and H = 150; (**d**) D = 300 mm and H = 300. The results of tests employing a hemispherical joint are plotted by the dashed lines, and for parallel plates—by the solid lines.

The experimentally determined variation of the natural frequency with compressive load is shown in Figure 9 (the data are plotted by markers, whereas the lines are only meant as a guide to the eye). Notably, for each shell geometry, the cylinders with higher buckling loads also tended to have higher natural frequencies. For example, specimens Cyl. 4 and 5, exhibiting the highest buckling loads for the shells with H = 200 and D =100 mm, also possessed the highest first natural frequencies (Figure 9a); Cyl. 3 with the lowest buckling load of cylinders with H = 300, D = 300 mm dimensions also had the lowest eigenfrequency among them (Figure 9d).

However, this is a tendency rather than an exact equivalence of specimen rankings in terms of the bucking load and eigenfrequency magnitude. So, among the shells with H = 400 and D = 100 mm, the specimens Cyl. 4N to 6N (loaded by parallel plates) had considerably higher buckling loads than Cyl. 1 to 3 (tested using a hemispherical joint), and this distinction also held for eigenfrequency magnitudes (Figure 9b). By contrast, the ranking of specimens in terms of the buckling load and natural frequency within each of these two subgroups did not coincide exactly.

The coefficient of correlation between the critical loads and the lowest eigenfrequencies for the unloaded shells with D = 100 mm amounted to 0.73 (H = 200 mm) and 0.67 (H = 400 mm), whereas for the shells with D = 300 mm, it was 0.97 (H = 300 mm) and 0.40 (H = 150 mm). Such a correlation apparently originates from the sensitivity of both the buckling load and the first natural frequency to imperfections and boundary conditions of thin-walled shells. It has been shown (see, e.g., [34]) that the presence of geometrical imperfections in shells reduce not only the critical load, but also the frequency of the lowest vibration mode. The sensitivity of the lowest eigenfrequency to actual boundary conditions has been used to evaluate the equivalent elastic restraints of shell edges in order to enable a more accurate prediction of the buckling load [18,23]. Stiffer restraints, naturally, also lead to greater critical load and natural frequency.

It is seen in Figure 9 that the reduction in the first natural frequency of cylinders under an increasing compressive load *P* is, in general, initially smooth under low loads, apart from a few cases. Namely, for Cyl. 6N (Figure 9b), the natural frequency in the unloaded state was actually lower than at low loads. Such an effect has also been reported before [28]. Conversely, for Cyl. 7 (Figure 9a) and Cyl. 4N (Figure 9b), the drop in frequency at the first loading step was markedly larger than the subsequent, smoother reduction of *f*_m_ at further load steps. Such an irregularity in the frequency response of these shells in the nominally unloaded state is likely to be caused by an interaction of the actual boundary conditions at *P* = 0 [21] and the stress state in the shells introduced by potting. The magnitude of such stresses was not assessed in the present work, but, notably, prestresses in cylindrical shells due to a minor mismatch of the shell and mounting ring geometry have been evaluated [11] and found to be capable of affecting the numerically estimated instability onset. The measured load-free spectra of the shells mentioned were excluded from a further VCT analysis, and the values of *f*_0_ were replaced by those obtained at the smallest non-zero axial load.

### 4.3. VCT-Based Prediction of the Critical Load

The linear buckling loads of shells needed for a VCT analysis were estimated by the respective finite-element models generated employing the ANSYS© Mechanical finite element analysis software. Linear elastic FEM analyses of cylindrical shells lacking any imperfections were performed using SHELL281 elements. The elasticity characteristics of UD plies reported in Table 1 were employed in calculations; the values of Young’s moduli as determined in compression tests were used. For 300-mm-diameter cylinders, exhibiting relatively large scatter in wall thickness *t* among shells (the coefficient of variation being ca. 0.2), the experimentally determined *t* value for each shell was implemented in the FEM model. For 100-mm-diameter cylinders, possessing smaller thickness scatter, the average value of ply thickness of 0.104 mm was used. Clamped boundary conditions were applied as follows: on the bottom edge of the shell, the condition of all edge displacement and rotation components being equal to zero was imposed. The boundary conditions of the upper edge differed only in that the vertical displacement of the edge nodes, equal for all the nodes, was applied to model the displacement-controlled axial compressive loading.

The predicted buckling loads *P*_VCT_, derived using the whole range of load–eigenfrequency data obtained and their relative deviations $\delta =\left|1-P_{VCT}/P_{b}\right|$ from the experimental ones are presented in Table 3. For the ease of reference, the VCT method [23] using Equation (2) for fitting the load–frequency data is further denoted by M1 and its empirical modification using Equation (7)—by M2. The largest deviation of VCT prediction by both the methods, ca. 35%, is seen for the specimen that was subjected to the lowest relative maximum load *P*/*P*_b_ = 0.65. For the rest of cylinders, vibration tests to higher relative maximum loads were performed, and the accuracy of the VCT-derived critical load estimates improved, as expected. Specifically, when eigenfrequency data up to *P*/*P*_b_ ≥ 0.87 were used, the maximum deviation of *P*_VCT_ was 29% for M1 method and 16% for M2. The average relative error of prediction by the method M2, amounting to 6.4%, was also lower than that by M1, 9.7%. Thus, the maximum and mean deviations of the VCT-predicted buckling load were both smaller for the empirical method M2.

**Figure 10:**
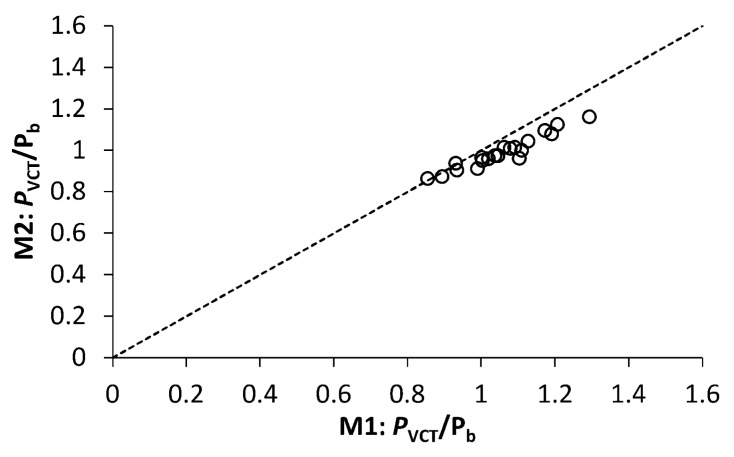
Ratio of VCT-estimated and experimental buckling loads, *P*_VCT_/*P*_b_, for method M2 as a function of that for method M1, using the maximum load *P*/*P*_b_ ≥ 0.87 in vibration tests.

The critical loads predicted by both methods appear to correlate rather closely, as seen in Figure 10 presenting the ratio of VCT-estimated and experimental buckling loads, *P*_VCT_/*P*_b_, for M2 as a function of that for M1. Such a correlation is likely to stem from the same implicit underlying criterion of instability, dp/df=0, shared by both methods, as demonstrated in Section 2. However, M2 produced more conservative estimates of the critical load than M1, and that feature became particularly pronounced for *P*_VCT_*/P*_b_ > 1, i.e., when the critical load was overestimated by the VCT. As can be inferred from Figure 10 and Table 3, *P*_VCT_ predicted by M1 deviates by no more than −14 to 29% from the experimental buckling load, while for prediction by M2, the deviation is within the range of −14 to 16%, when vibration data up to the maximum load *P*/*P*_b_ ≥ 0.87 are used.

**Figure 11:**
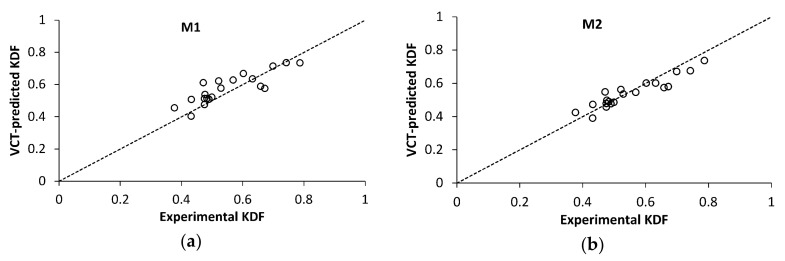
VCT-predicted knock-down factor, using maximum load in vibration tests *P*/*P*_b_ ≥ 0.87, as a function of the experimental KDF for VCT method: (**a**) M1; (**b**) M2.

Notably, both VCT methods tend to slightly underestimate the critical load for shells with large experimental KDF values, as seen in Figure 11. It follows from the previous research, Figure 1a, and present results, Figure 11a, that M1 yields a conservative prediction for the experimental KDF exceeding about 0.65. For M2, the data in Figure 11b suggest that the transition to a conservative prediction takes place at a slightly lower KDF of ca. 0.55. Such a feature of the VCT-predicted critical loads appears beneficial for shells with a high fabrication quality leading to a high KDF, as would be expected in the aerospace sector. For such high-quality shells, the VCT provides not only close, but also conservative buckling load estimates.

Both the VCT methods considered are robust to using either the natural frequency of an unloaded specimen or of a specimen loaded with a small axial force *P* ≤ 0.1*P*_b_ as the starting point *f*_0_ for characterization of the natural frequency dependence on the axial load: the corresponding mean values of *δ* were 7.5 and 13.1% for M1, and 6.0 and 6.9% for M2 at *P*/*P*_b_ ≥ 0.87. This is in agreement with a previous finding that the VCT prediction by M1 is not affected by the absence of data for the first natural frequency of an unloaded shell [21]. Similarly, the geometrical characteristics of the tested cylinders have little effect on the prediction accuracy, Table 4.

Method M2 is also virtually robust with respect to variations in the mounting and loading methods of specimens, as demonstrated by the minor difference in the average accuracy of prediction among the three combinations of shell edge support and load introduction methods, Table 5.

**Figure 12:**
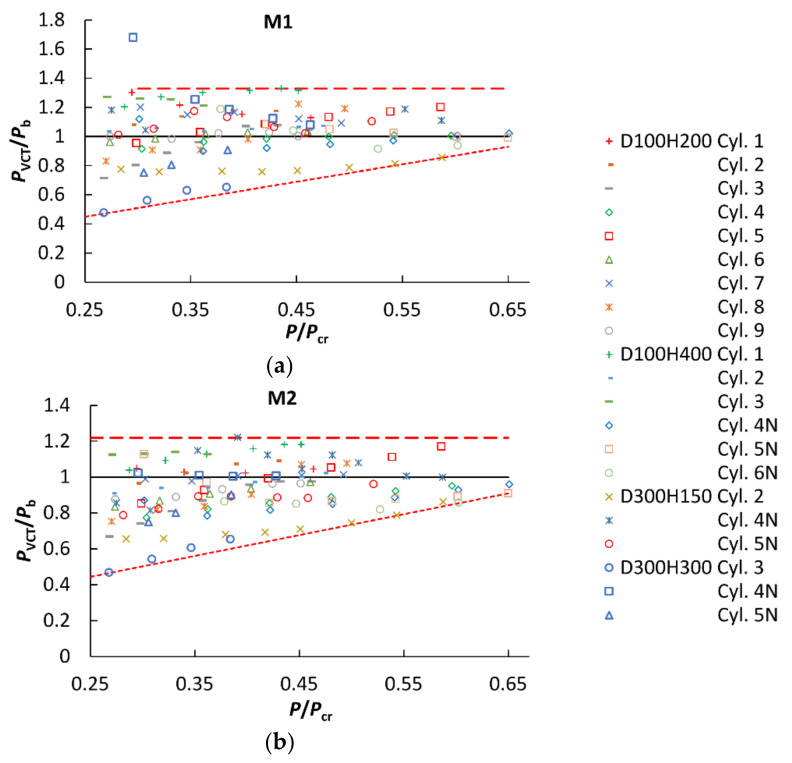
Predicted buckling load *P*_VCT_/*P*_b_ as a function of the highest axial load used in vibration tests, *P*/*P*_cr_, for *P*/*P*_cr_ > 0.25, using the VCT method: (**a**) M1; (**b**) M2.

For eventual practical applications of VCT, it is of interest to consider the maximum axial load to be used for evaluating the reduction of the natural frequency in terms of the linear buckling load *P*_cr_. Analyzing the incremental evolution of the VCT-predicted critical load as a function of the axial load, natural frequency variation up to which is used in VCT analysis, *P*_VCT_ = *P*_VCT_(*P*), spurious results were obtained for some of specimens at relatively low numbers of loading steps and, hence, at low *P* values. The minimum number of load–frequency data points allowing the evaluation of the parameters of VCT relations Equations (2) and (7) is, naturally, equal to the number of equation parameters, namely, three. They correspond to the unloaded state of the shell and two subsequent loading steps. Using such a limited dataset in the analysis occasionally led to physically unreasonable results, such as a negative predicted buckling load, *P*_VCT_ < 0, or *ξ* ^2^ < 0 (as calculated by Equation (4) or (8)), which eventually disappeared when a greater number of load steps, corresponding to a higher load, were used. These findings apparently agree with the suggestions concerning load steps [28] and the maximum load level [21] in vibration tests. It is recommended to use a greater number of experimental points, at least 15 [28], to reach a reliable buckling prediction by the VCT. However, increasing the maximum load level was found to be even more effective for reducing the deviations *δ* than the number of load steps [21]. Based on the current dataset, we selected a cut-off load level at *P*/*P*_cr_ = 0.25, starting from which to consider the accuracy of VCT. This corresponded to at least four to seven load levels in vibration tests. The predicted buckling load *P*_VCT_/*P*_b_ as a function of the highest axial load used in vibration tests, expressed as a fraction of the linear buckling load, *P*/*P*_cr_, is shown in Figure 12 for all the specimens tested.

It is seen that, for both VCT methods considered, the lower bound of the normalized predicted buckling load *P*_VCT_/*P*_b_ increased almost linearly with the maximum load used in vibration tests (the dotted lines in Figure 12), approaching unity at *P*/*P*_cr_ = 0.65 for method M1 and 0.9 for method M2. By contrast, the upper bound of the predicted buckling load data had no clear dependence on the axial load up to *P*/*P*_cr_ ~ 0.6 for M2, the greatest overestimation of the experimental buckling load being *P*_VCT_/*P*_b_ = 1.22 (the dashed line in Figure 12b). Similarly, method M1 overestimated the experimental buckling load by, at most, the factor of *P*_VCT_/*P*_b_ = 1.33 for *P*/*P*_cr_ > 0.3, as marked by the dashed line in Figure 12a. It can be inferred from these results that, for the VCT-predicted buckling load, a knock-down factor of about 0.81 has to be applied if M2 is used on load–eigenfrequency data up to loads *P* ≥ 0.25 *P*_cr_, whereas an even smaller knock-down factor or a larger load range is needed for M1.

## 5. Conclusions

The vibration correlation technique for nondestructive evaluation of the critical load of thin-walled imperfection-sensitive shells has been applied for prediction of the buckling load of CFRP cylinders. The buckling loads in axial compression have been determined experimentally for 21 unstiffened cylindrical CFRP shells with identical lay-ups, four different geometries, two shell mounting techniques, and two load introduction methods. The modal behavior of the CFRP cylindrical shells under axial compression was investigated by exciting the vibrations by a loudspeaker, thus determining the variation of the first natural frequency of vibrations with the applied load.

It was found that the prediction accuracy of the buckling load using either VCT approach proposed by Arbelo at al. [23] or its empirical modification was virtually insensitive to shell geometry and mounting and loading methods. Moreover, the VCT methods also appeared robust with respect to a lack of natural frequency data for an unloaded shell, caused, e.g., by the need for a preload to reliably fix the shell in the test rig. Both VCT methods tended to slightly underestimate the critical load for shells with relatively large experimental KDF values thus providing not only close, but also conservative estimates of the limit load for high-quality shells.

The modified VCT method yielded an average relative error of prediction of 6.4% when natural frequencies at loads exceeding ca. 0.9 of the buckling load were used. Upon reframing the VCT results in terms of the critical load of a perfect shell, it appeared that the vibration response under loads exceeding 0.25 of the linear buckling load enabled a successful application of VCT, but a knock-down factor of ca. 0.81 had to be applied. Further elaboration of the VCT approach is warranted to reduce the load range needed for eigenfrequency monitoring and to increase the prediction accuracy of the buckling load.

## Figures and Tables

**Table 1:** Elastic properties of a Unipreg^®^ UD composite [25].

Longitudinal Young’s Modulus *E*_11_, GPa		Transverse Young’s Modulus *E*_22_, GPa		In-Plane Shear Modulus *G*_12_, GPa	Poisson’s Ratio *ν*_12_
Tension	Compression	Tension	Compression		
116.4	91.7	6.7	6.4	3.6	0.34

**Table 2:** Buckling loads of CFRP shells.

Shell Geometry			Load Introduction	Critical Load *P*_b_, kN
Diameter D, mm	Height H, mm	Wall Thickness *t*, mm		
100	200	0.294 (0.001) ^1^	Hemispherical joint	3.05 (0.21) ^1^
		0.273 (0.010)	Parallel plates	3.65 (0.49)
	400	0.298 (0.005)	Hemispherical joint	2.97 (0.39)
		0.355 (0.019)	Parallel plates	4.96 (0.29)
300	150	0.359 (0.014)	Parallel plates	5.63 (0.21)
	300	0.336 (0.046)		3.88 (0.51)

^1^ Standard deviation.

**Table 3:** Comparison of experimental and VCT-predicted buckling loads.

Cylinder Diameter and Height, mm	Loading	Cylinder Number	Buckling Load *P*_b_, kN	Load Range in Vibration Tests		VCT-Predicted Buckling Load			
						M1		M2	
				Min. *P*/*P*_b_	Max. *P*/*P*_b_	*P*_VCT_, kN	Relat. Error *δ*, %	*P*_VCT_, kN	Relat. Error *δ*, %
D = 100 H = 200	Hemi- spherical joint	01	3.20	0.05	0.97	3.61	12.9	3.34	4.5
		02	2.90	0.05	0.99	3.40	17.4	3.17	9.3
	Parallel plates	03	3.35	0	0.93	3.50	4.7	3.26	2.5
		04	4.24	0	0.94	4.26	0.4	4.03	5.0
		05	4.42	0	0.89	3.95	10.5	3.85	12.8
		06	3.29	0	0.94	3.41	3.7	3.2	2.6
		07	3.55	0.09	0.93	3.87	9.1	3.59	1.2
		08	3.50	0	0.95	4.17	19.3	3.77	7.7
		09	3.19	0	0.95	3.19	0.2	3.07	3.5
D = 100 H = 400	Hemi- spherical joint	01	3.15	0.08	0.97	4.08	29.4	3.66	16.2
		02	3.23	0.04	0.99	3.43	6.3	3.27	1.2
		03	2.52	0.08	0.96	3.04	20.8	2.83	12.4
	Parallel plates	04N	4.67	0.08	0.93	4.77	1.9	4.48	4.2
		05N	4.96	0	0.88	4.91	0.9	4.51	9.0
		06N	5.26	0.10	0.95	4.9	6.8	4.92	6.4
D = 300 H = 150	Parallel plates	02	5.61	0	0.87	4.8	14.4	4.84	13.8
		04N	5.85	0	0.98	6.49	11.0	5.84	0.1
		05N	5.44	0	0.92	6.0	10.4	5.22	4.0
D = 300 H = 300	Parallel plates	03	3.29	0	0.65	2.14	34.9	2.15	34.6
		04N	4.24	0	0.97	4.57	8.0	4.27	0.7
		05N	4.11	0	0.89	3.84	6.5	3.71	9.8

**Table 4:** Accuracy of VCT prediction (at maximum *P*/*P*_b_ ≥ 0.87) as a function of cylinder geometry.

Relative Length Parameter of Shell $\omega =H/\sqrt{R\cdot t}$	Mean Relative Error of Prediction *δ*, %	
	M1	M2
21	11.9	6.0
42	7.3	5.3
54	8.7	5.5
99	11.0	8.2

**Table 5:** Accuracy of VCT prediction (at maximum *P*/*P*_b_ ≥ 0.87) for different loading and boundary conditions of cylinders.

Loading and Boundary Conditions	Mean Relative Error of Prediction *δ*, %	
	M1	M2
Steel ring, hemispherical joint	17.4	8.7
Steel ring, parallel plates	5.8	5.5
Potted, parallel plates	10.1	5.7
